# Erector spinae plane block versus transversus abdominis plane block for analgesia after cesarean section: a systematic review and meta-analysis

**DOI:** 10.1016/j.bjane.2025.844606

**Published:** 2025-03-09

**Authors:** Mariana AbdElSayed Mansour, Saeed Baradwan, Ahmed Abdelaziz Shama, Mohamed Ali Mahmoud, Ayman Salah Abouelnour, Ayman Mohamed AbdelWahed Mohamed, Ahmed Fathi Hassan Elkhouly, Abdelkarem Hussiny Ismail Elsayed, Zaky Ftouh Rashed, Ahmed Mohamed Abdelhakim, Mrooj Mabruk Almutairi, Mohamed A. Lotfy, Ahmed Goda Ahmed

**Affiliations:** a Beni-Suef University, Faculty of Medicine, Department of Anesthesiology, Surgical Intensive Care and Pain Management, Beni-Suef, Egypt; b King Faisal Specialist Hospital and Research Center, Department of Obstetrics and Gynecology, Jeddah, Saudi Arabia; c Tanta University, Faculty of Medicine, Department of Anesthesiology, Surgical Intensive Care and Pain Management, Tanta, Egypt; d Al-Azhar University, Faculty of Medicine, Department of Anesthesiology, Surgical Intensive Care and Pain Management, Assiut, Egypt; e Al-Azhar University, Faculty of Medicine, Department of Anesthesiology, Surgical Intensive Care and Pain Management, Cairo, Egypt; f Al-Azhar University, International Islamic Center for Population Studies and Research, Department of Anesthesiology, Surgical Intensive Care and Pain Management, Cairo, Egypt; g Al Maarefa University, College of Applied Sciences, Department of Anesthesia, Riyadh, Saudi Arabia; h Cairo University, Faculty of Medicine, Kasralainy, Cairo, Egypt; i King Abdulaziz University, Faculty of Medicine, Jeddah, Saudi Arabia

**Keywords:** Anesthesia, Cesarean section, Meta-analysis, Obstetrics, Systematic review

## Abstract

**Background:**

Peripheral abdominal nerve blocks are key components of multimodal analgesia, enhancing recovery after cesarean sections. This systematic review and meta-analysis aimed to assess analgesic efficacy of Erector Spinae Plane Block (ESPB) versus Transversus Abdominis Plane Block (TAPB) under ultrasound guidance following Cesarean Section (CS) under spinal anesthesia.

**Methods:**

A comprehensive search was conducted across PubMed, Scopus, Cochrane Library, and ISI Web of Science to identify relevant trials. The inclusion criteria followed the PICOS framework: Population (women undergoing elective cesarean delivery), Intervention (ESPB), Comparator (TAPB), Outcomes (postoperative pain, opioid consumption, analgesic duration, and satisfaction), and Study Design (randomized controlled trials).

**Results:**

Seven RCTs (380-patients) met the inclusion criteria. The ESPB group had significantly lower postoperative pain scores at rest and during movement, reduced 24-hour opioid consumption (MD = -2.62 MME; 95% CI -4.11 to -1.13; p = 0.006), and longer analgesic duration (SMD = 1.77; 95% CI 1.11 to 2.44; p < 0.001) than the TAPB group. Patient satisfaction was also significantly higher in the ESPB group (OR = 4.75; 95% CI 2.26 to 9.99; p < 0.001). While most outcomes demonstrated low heterogeneity, significant variability was observed in analgesic duration (I^2^ = 83%), requiring cautious interpretation.

**Conclusions:**

The ESP block offers superior pain relief, reduces opioid use, and enhances satisfaction compared to the TAP block in cesarean sections. These findings suggest that the implementation of the ESP block in postoperative analgesia protocols could significantly improve patient outcomes, potentially leading to enhanced recovery and reduced reliance on opioids.

## Introduction

In recent decades, the percentage of births delivered via Cesarean Section (CS) has risen, currently surpassing 32%.^1^ Women often experience moderate to severe pain following a cesarean delivery, with over 10% developing persistent pain lasting beyond 3 to 6 months.^2^^,^^3^ Proper pain management after the procedure is crucial for promoting early recovery and mobility, minimizing the negative consequences of pain, and facilitating quicker bonding between mother and baby.^4^ Insufficient control of postoperative pain can result in slower recovery, ongoing pain, higher reliance on opioids, decline in quality of life, and a higher likelihood of postpartum depression.^5^^,^^6^

Multimodal analgesia for managing postoperative pain following cesarean deliveries involves the use of intrathecal, epidural, and/or systemic opioids, in addition to regional techniques such as truncal blocks.^7^^,^^8^ Although intrathecal morphine offers superior pain relief, it may lead to various side effects, including nausea, severe itching, respiratory depression, urinary retention, and drowsiness.^9^ As a result, regional anesthesia has become increasingly preferred.^10^ Truncal blocks, such as Transversus Abdominis Plane (TAP) block and Erector Spinae Plane (ESP) block, are among the most frequently employed methods for managing postoperative pain following cesarean sections.^11^

The Transversus Abdominis Plane Block (TAPB) is a modern technique used for pain control in lower abdominal surgeries. It involves injecting a local anesthetic into the transversus abdominis plane using either anatomical landmarks or ultrasound guidance.^12^ The transversus abdominis plane is situated between the internal oblique and transversus abdominis muscles, where the spinal nerve branches responsible for sensory innervation of the abdominal wall, muscles, and parietal peritoneum are located.^13^ Meta-analyses indicate that TAP blocks lower pain levels and decrease the need for opioids, leading various international guidelines to endorse their use as analgesic adjuncts following cesarean sections.^14^^,^^15^ However, while TAPB effectively relieves somatic pain, it has limited impact on visceral nerves.^16^

The Erector Spinae Plane Block (ESPB) is a recent regional anesthetic method introduced in 2016, offering a viable option for pain management in various surgical scenarios.^17^ Forero et al. were the first to document the ESPB, highlighting its potential to not only alleviate somatic pain but also to address visceral pain by blocking the ventral, dorsal, and communicating spinal nerve branches.^18^ This technique can be conducted utilizing clearly identifiable ultrasound landmarks, ensuring a higher level of safety.^19^ Studies indicate that utilizing an ESP block during cesarean delivery leads to decreased pain levels and less reliance on opioids compared to placebo controls.^20^^,^^21^

Research comparing the postoperative analgesic effectiveness between ESP and TAP blocks following CS has yielded conflicting results. Some studies indicated that the ESP block provides superior pain management compared to the TAP block,^22^^,^^23^ while others suggested that both techniques are equally effective in managing pain.^24^^,^^25^ This inconsistency in the literature highlights a clinical gap in determining the optimal regional anesthesia technique for cesarean sections. Given the importance of effective pain management in improving recovery outcomes and reducing opioid consumption after CS, we conducted this meta-analysis to assess and compare the analgesic effectiveness of ESP block versus TAP block specifically for patients undergoing cesarean sections.

## Materials and methods

This systematic review and meta-analysis adhered to the eligibility criteria outlined by the Preferred Reporting Items for Systematic Reviews and Meta-Analyses (PRISMA) recommendations.^26^ All data included in this review were sourced from published studies, which eliminated the need for ethical approval. Additionally, this meta-analysis did not receive any funding.

### Search strategy

We conducted an extensive search for relevant articles in multiple databases including PubMed, Scopus, Cochrane Library, and ISI Web of Science from their inception to August 2024, without limiting by language or publication year. Our search strategy utilized the following keywords: [“Erector spinae plane block” OR “Erector spinae plane nerve block” OR “ESP block” OR “ESPB”] AND [“Transversus abdominis plane block” OR “Transabdominal abdominis plane block” OR “TAP block” OR “TAPB”] AND [“Cesarean section” OR “Cesarean delivery” OR “Cesarean” OR “C-section” OR “Caesarean” OR “Caesarean section” OR “Caesarean delivery”]. The literature search was carried out independently by two authors, and any differences were resolved through mutual agreement.

### Study selection

We chose studies that conformed to the following PICOS criteria: 1) Patients: women scheduled for elective cesarean section under spinal anesthesia, 2) Intervention: ultrasound-guided ESP block, 3) Control: ultrasound-guided TAP block, 4) Outcomes: postoperative pain levels, postoperative opioid consumption, and duration of the block, and 5) Study design: Randomized Controlled Trials (RCTs). The specifics regarding the type, dosage, and volume of local anesthetics, as well as the use of adjuvants in each group, were not factors for the eligibility of the studies. We did not include trials that compared ESP blocks to TAP blocks for surgical procedures other than cesarean delivery. Furthermore, we excluded review articles, opinion articles, letters, editorials, retrospective studies, non-randomized controlled trials, and studies that focused on outcomes outside our specific areas of interest. We excluded non-RCTs to ensure the inclusion of studies with the highest level of methodological rigor and to minimize potential sources of bias. Two reviewers assessed the titles and abstracts of the potential publications independently. The full texts of the articles that were initially identified and appeared to meet the eligibility criteria were subsequently re-evaluated prior to making the final decision. In instances of disagreement, a third reviewer was consulted to reach a conclusion.

### Data extraction

Three independent reviewers gathered relevant data from the selected studies using a uniform data collection sheet. Any inconsistencies were addressed through discussions among the authors. The data collected comprised the names of the first author, publication year, study groups, study location, sample size, maternal age, Body Mass Index (BMI), surgery duration, types and dosages of local anesthetics employed for both blocks, spinal anesthesia protocols, and main findings. The primary outcome measured was pain severity at rest and during movement or coughing, assessed at 4, 8, 12, and 24 hours after cesarean delivery using the Visual Analog Scale (VAS). Secondary outcomes included the amount of opioids consumed, duration of the block in hours, and patient satisfaction with analgesia after cesarean section. The duration of block was identified as the period from when the block was administered to the moment the patient first requested pain relief post-cesarean section. All opioids administered for postoperative pain relief were converted into Morphine Milligram Equivalent (MME) units based on the standardized conversion tables from the British National Formulary. The Likert verbal rating scale was utilized to evaluate patient satisfaction. Certain outcomes did not provide the necessary mean and standard deviation values, so these were derived from alternative parameters such as medians, ranges (minimum–maximum), or interquartile ranges, as outlined by Wan et al.^27^

### Risk of bias (quality) assessment

The quality of the studies included was assessed using the Cochrane Risk of Bias Tool.^28^ This assessment focused on several key areas: 1) Random sequence generation; 2) Allocation concealment; 3) Blinding of participants and personnel; 4) Blinding of outcome assessment; 5) Incomplete outcome data; 6) Selective reporting; and 7) Other forms of bias. Each area was categorized as low risk, high risk, or unclear risk. Two authors carried out the evaluation independently, and any disagreements were settled through discussion.

We utilized the Grading of Recommendations, Assessment, Development, and Evaluation (GRADE) approach to systematically assess the quality of evidence.^29^ This assessment considered factors such as risk of bias, inconsistency, indirectness, imprecision, and publication bias. Evidence quality was categorized as high, moderate, low, or very low by two review authors working independently, with disagreements resolved through discussion.

### Statistical analysis

One author input the data into Review Manager 5.4.0, while another author verified it for statistical analysis. For continuous data, including pain severity scores at various time points, block duration, and total opioid usage post-cesarean delivery, the analysis utilized Mean Difference (MD) or Standardized Mean Difference (SMD) with a 95% Confidence Interval (95% CI). In contrast, dichotomous outcomes, such as patient satisfaction with analgesia, were assessed using Odds Ratio (OR) along with a 95% Confidence Interval (95% CI). A p-value of less than 0.05 was established as the threshold for statistical significance.^30^ The heterogeneity of the studies was evaluated using the I-squared (I^2^) statistic and the chi-square test. An I^2^ value of < 50% and a p-value > 0.1 suggested that there was no significant heterogeneity. Conversely, an I^2^ value of > 50% and a p-value of < 0.1 indicated notable heterogeneity. We employed a random-effects model for the meta-analysis, independent of the I^2^ results.^31^ A sensitivity analysis was conducted for the correction of heterogeneous outcomes in which we excluded one study at a time “one-out sensitivity analysis” and evaluated the impact of removing each of the studies on the summary results and between-study heterogeneity. Given that fewer than 10 studies were included in this systematic review and meta-analysis, the assessment of publication bias using funnel plots was not conducted.^32^^,^^33^

## Results

### Results of the literature search and Characteristics of the included studies

**Figure 1:**
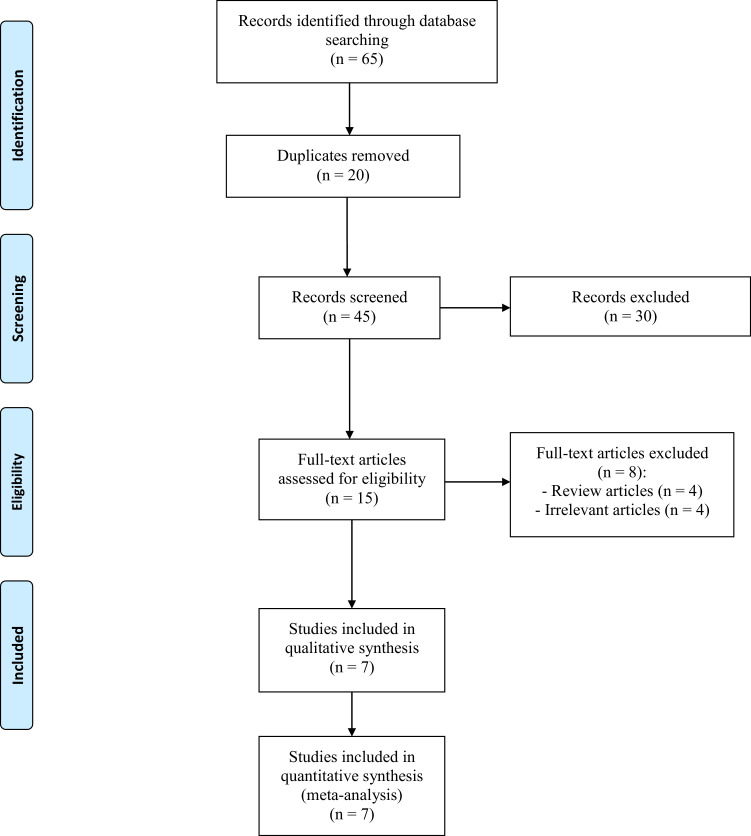
PRISMA flow diagram.

The PRISMA flow diagram (Fig. 1) illustrates the study selection process for this research. Initially, 65 studies were found through database searches. After reviewing the titles and abstracts, 15 articles were selected for full-text evaluation. From these, eight articles were excluded, leaving seven studies included for both qualitative and quantitative analysis. These seven studies^22^, ^23^, ^24^, ^25^^,^^34^, ^35^, ^36^ were Randomized Controlled Trials (RCTs) that fulfilled our inclusion criteria. All included studies received appropriate ethical approval, with patient consent obtained as required by the respective institutional review boards.

Each trial compared the effectiveness of ESPB and TAPB for postoperative pain relief after cesarean delivery, with all using ultrasound guidance for both blocks. All women underwent cesarean sections using intrathecal spinal anesthesia with hyperbaric bupivacaine, with doses ranging from 9 to 12.5 mg, administrated with or without fentanyl. The sample sizes for the included RCTs ranged from 24 to 66 participants, with an overall total of 380 individuals involved. Of these, 190 participants were allocated to the ESPB group, while the remaining 190 were placed in the TAPB group. There was variability in the dosages, types, drug combinations, and adjuvants used with local anesthetics across the studies; however, all studies administered the same dose and type of medication for both blocks. In both groups, bupivacaine was the main local anesthetic utilized in five trials,^23^^,^^24^^,^^34^, ^35^, ^36^ while ropivacaine was used in two trials.^22^^,^^25^ The included studies took place in several countries, including Egypt,^23^^,^^34^^,^^35^ India,^22^^,^^25^ Turkey,^36^ and South Africa.^24^ Table 1 outlines the characteristics of the studies involved.

**Table 1:** Characteristics of the included studies.

Study ID	Study location	Study groups	Sample size	Maternal age (years)	Body mass index (BMI)	Duration of surgery (min)	Local anesthetics type and dosage for both blocks	Spinal anesthesia protocol	Main findings
Eksteen et al.,^24^ 2024	South Africa	ESPB group	33	31 ± 5.9	28.6 ± 4.4	45±17	20 mL Bupivacaine 0.25%	Hyperbaric bupivacaine (9 mg) with fentanyl (10 mcg) intrathecally	ESPB and TAPB were similar in analgesic efficacy and postoperative opioid administration after cesarean section.
		TAPB group	33	30.5 ± 5	30.1 ± 3.2	45±19			
Balata et al.,^34^ 2023	Egypt	ESPB group	12	27.92 ± 5.76	25.61 ± 1.86	NA	15 mL Bupivacaine 0.25%	Hyperbaric bupivacaine (12.5 mg) intrathecally.	ESPB provided extended analgesia with appreciably lower opioid requirements and associated with lower complications and higher patient satisfaction compared to TAPB after cesarean section.
		TAPB group	12	28.75 ± 5.5	26.57 ± 1.62	NA			
Reddy et al.,^25^ 2023	India	ESPB group	25	29.8 + 3.5	26.4 + 2.9	NA	20 mL Ropivacaine 0.2%	Hyperbaric bupivacaine (9 mg) with fentanyl (10 mcg) intrathecally	ESPB and TAPB provide similar analgesia with comparable opioid consumption and no difference in pain scores in the first 24 hours after cesarean delivery.
		TAPB group	25	29.2 + 3.9	27.4 + 3.9	NA			
Yilmaz & Erol,^36^ 2023	Turkey	ESPB group	30	31.5 ± 5.01	23.35 ± 2.6	50.47 ± 11.07	20 mL Bupivacaine 0.25%	Hyperbaric bupivacaine (10 mg) intrathecally	ESPB is more effective regarding pain scores, opioid consumption, and patient satisfaction compared to TABP after cesarean section.
		TAPB group	30	31.13 ± 5.94	23.94 ± 2.5	50.3 ± 12.56			
Elshafay et al.,^35^ 2022	Egypt	ESPB group	30	28.43 ± 2.9	21.1 ± 2.6	50.33 ± 5.5	20 mL Bupivacaine 0.25%	Hyperbaric bupivacaine (12.5 mg) intrathecally	ESPB was associated with longer duration of analgesia, lower pain scores, and lower total opioid consumption during the first 24 hours after cesarean section compared to TAPB.
		TAPB group	30	27.87 ± 3.3	21.5 ± 2.4	49.27 ± 5.6			
Boules et al.,^23^ 2020	Egypt	ESPB group	30	27.1 ± 6	26.7 ± 4.2	NA	20 mL Bupivacaine 0.25%	Hyperbaric bupivacaine (12 mg) intrathecally	ESPB provides more effective pain relief, has a longer duration of analgesic action, prolongs time to first analgesic requirement, and is associated with less opioid consumption compared to TABP after cesarean section.
		TAPB group	30	28.9 ± 5.5	26.3 ± 5.8	NA			
Malawat et al.,^22^ 2020	India	ESPB group	30	28 ± 3	22.7 ± 5	45 ± 10	20 mL Ropivacaine 0.2%	Hyperbaric bupivacaine (12.5 mg) intrathecally	ESPB provided prolonged analgesia with a significant decrease in analgesic requirement compared to TAPB and can be used as a standard technique for post-cesarean analgesia.
		TAPB group	30	30±3	23.9 ± 4	44 ± 9			

### Risk of bias of included studies and quality of evidence

**Figure 2:**
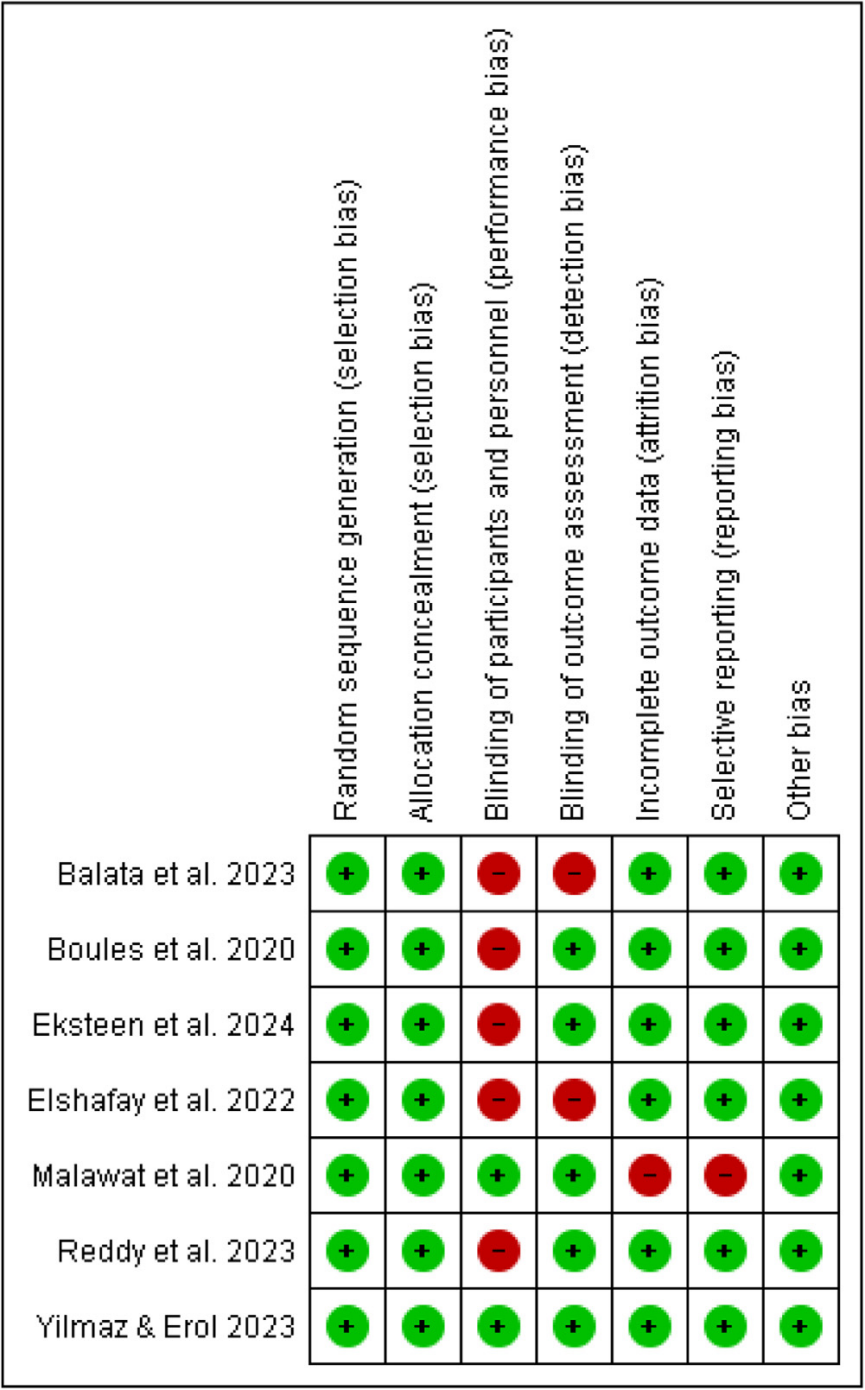
Risk of bias summary of the included RCTs.

Figure 2 provides a summary of the bias risk for the RCTs considered in the analysis. All studies demonstrated a low risk of bias concerning random sequence generation, allocation concealment, and other potential bias sources. In six studies,^23^, ^24^, ^25^^,^^34^, ^35^, ^36^ there was a low risk of bias related to incomplete outcome data and selective reporting, while one study showed a high risk in this area.^22^ Two studies^22^^,^^36^ had a low risk of bias for participant and personnel blinding, whereas five studies had a high risk.^23^, ^24^, ^25^^,^^34^^,^^35^ For outcome assessment blinding, five studies were assessed as low risk,^22^, ^23^, ^24^, ^25^^,^^36^ and two studies were deemed high risk.^34^^,^^35^ The overall strength of evidence for our selected outcomes, as evaluated by the GRADE approach, was deemed “moderate”.

### Primary outcomes: postoperative pain scores

**Figure 3:**
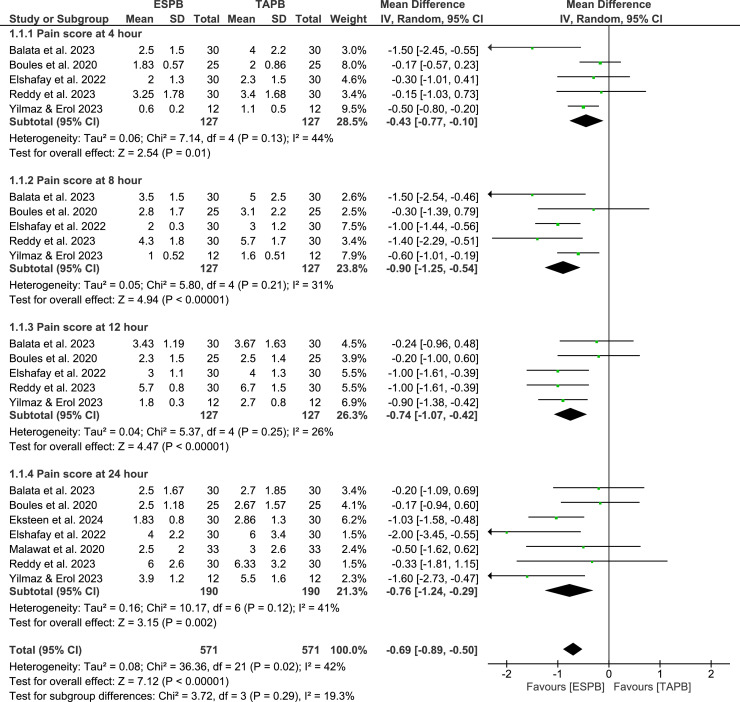
Postoperative pain scores at rest.

**Figure 4:**
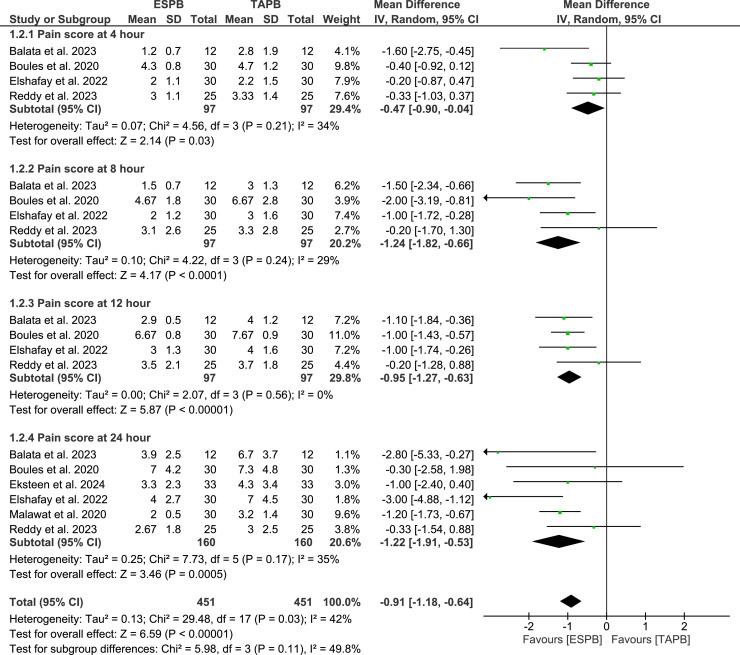
Postoperative pain scores on movement.

The meta-analysis results indicated that the ESPB group experienced significantly lower postoperative pain scores, measured by VAS, at various time intervals compared to the TAPB group: at rest at 4h (MD = -0.43; 95% CI -0.77 to -0.10; p = 0.01; I^2^ = 44%), 8h (MD = -0.90; 95% CI -1.25 to -0.54; p < 0.001; I^2^ = 31%), 12h (MD = -0.74; 95% CI -1.07 to -0.42; p < 0.001; I^2^ = 26%), and 24h (MD = -0.76; 95% CI -1.24 to -0.29; p = 0.002; I^2^ = 41%) (Fig. 3); and at movement at 4h (MD =-0.47; 95% CI -0.90 to -0.04; p = 0.03; I^2^ = 34%), 8h (MD = -1.24; 95% CI -1.82 to -0.66; p < 0.001; I^2^ = 29%), 12h (MD = -0.95; 95% CI -1.27 to -0.63; p < 0.001; I^2^ = 0%), and 24h (MD = -1.22; 95% CI -1.91 to -0.53; p = 0.005; I^2^ = 35%) (Fig. 4).

### Secondary outcomes: postoperative opioid consumption, block duration, and satisfaction

**Figure 5:**
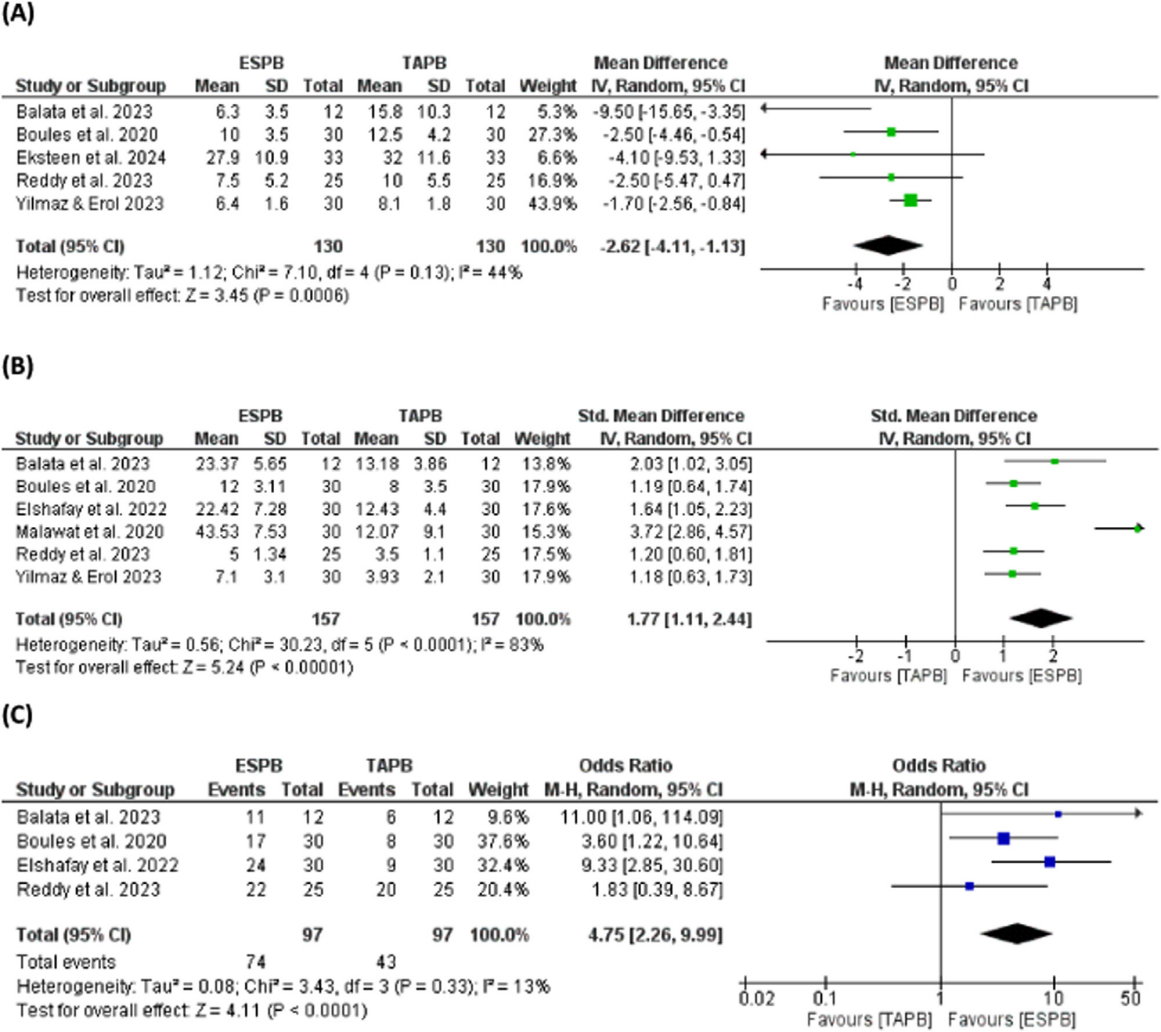
(A) Postoperative opioid consumption. (B) Duration of the block. (C) Patient satisfaction.

Five trials examined postoperative opioid usage, revealing that the ESPB group reduced opioid consumption 24 hours after CS compared to the TAPB group (MD = -2.62 MME; 95% CI -4.11 to -1.13; p = 0.006; I^2^ = 44%), as illustrated in Figure 5A.

Six trials investigated the duration of block following CS. The ESPB group demonstrated a longer duration of analgesic block compared to the TAPB group (SMD = 1.77; 95% CI 1.11 to 2.44; p < 0.001), as shown in Figure 5B. These studies showed heterogeneity (p < 0.001; I^2^ = 83%). By excluding one trial,^22^ the reported heterogeneity was resolved (p = 0.47; I^2^ = 0%), indicating a prolonged analgesic effect among the ESPB group (SMD = 1.35; 95% CI 1.0 to 1.63; p < 0.001).

Four trials assessed patient satisfaction 24 hours following CS. A higher percentage of patients in the ESPB group reported being greatly satisfied with their pain relief (76%) compared to those in the TAPB group (44%) (OR = 4.75; 95% CI 2.26 to 9.99; p < 0.001; I^2^ = 13%) (Fig. 5C).

## Discussion

Optimal post-cesarean pain management should prioritize rapid recovery, mobility, and breastfeeding while minimizing systemic side effects, which can be safely and effectively achieved with regional anesthesia techniques. This meta-analysis revealed that the ESP block significantly reduced pain scores at all assessed time points compared to the TAP block, reinforcing its efficacy in postoperative analgesia. Furthermore, the ESP block significantly reduced opioid use in the first 24 hours, provided longer analgesia, and resulted in higher patient satisfaction compared to the TAP block.

Boules et al. assessed the effectiveness of ESPB with TAPB for postoperative analgesia in patients undergoing elective CS.^23^ They found that the ESPB group experienced a longer duration of analgesia and a delayed first request for pain relief compared to the TAPB group. VAS pain scores, both at rest and during coughing, were notably lower in the ESPB group at 8 and 12 hours following surgery, though no significant differences were noted at 4 and 24 hours.^23^ Furthermore, total opioid use within the first 24 hours was significantly reduced in the ESPB group. Maternal satisfaction levels were similar across both groups, with no adverse effects reported.^23^ Two other trials also indicated lower VAS pain scores at various time points and longer analgesic duration following CS in the ESPB group compared to the TAPB group, along with reduced total opioid consumption and higher patient satisfaction in the ESPB group.^34^^,^^35^

Another study revealed that the initial administration of analgesics following cesarean delivery was significantly earlier in the TAPB group compared to the ESPB group.^36^ At 2, 4, and 8 hours post-operation, VAS pain scores were lower in the ESP block group, with no differences observed at 12 and 24 hours.^36^ Additionally, total opioid use within 24 hours was significantly reduced in the ESP block group, and both patient and surgeon satisfaction scores were higher compared to the TAP block group.^36^ Malawat et al. also noted that patients receiving the ESP block had a longer duration before their first request for analgesics, reported lower pain levels at rest and during movement, and consumed less total diclofenac than those in the TAP block group.^22^

On the contrary, Eksteen et al. reported no significant differences in total morphine usage, pain levels either at rest or during movement, and overall satisfaction with analgesia between the ESP and TAP blocks after cesarean sections under spinal anesthesia.^24^ They noted that ESP blocks took longer to perform and concluded that they did not provide significant additional visceral pain relief.^24^ Similarly, Reddy et al. reported no differences in VAS pain scores, 24-hour tramadol usage, or patient satisfaction between the two blocks, though the time until the first request for analgesia was significantly longer in the ESPB group.^25^ In a prior systematic review and meta-analysis, Junior et al. focused on just three trials to evaluate the analgesic effects of ESPB following cesarean deliveries.^37^ They concluded that ESPB did not reduce postoperative pain scores in comparison to other methods. Nevertheless, it was associated with decreased tramadol usage and extended duration of the analgesic blockade.^37^

Hamed et al. assessed the efficacy of ESPB compared to Intrathecal Morphine (ITM) for analgesia after elective CS under spinal anesthesia.^38^ No significant differences were reported regarding postoperative pain scores among both groups. However, they reported that total tramadol consumption in the first 24h was significantly higher in ITM group than in the ESPB group. The time to the first analgesic request was significantly shorter in the ITM group.^38^ An observational study recently evaluated the efficacy of ESPB and Quadratus Lumborum Block (QLB) for postoperative analgesia following cesarean delivery. The study found no significant differences between the two groups in total morphine consumption within the first 24 hours, postoperative pain scores, or the time to the first dose of morphine. However, the ESPB group required fewer rescue doses of morphine compared to the QLB group.^39^ Additionally, two recent trials concluded that ESPB and QLB were similarly effective in providing postoperative analgesia as part of multimodal analgesia after CS, with comparable outcomes in total tramadol consumption, pain scores, and the duration of analgesia.^40^^,^^41^

Our findings can be attributed to the different mechanisms and action locations of the two block types. The ESP block provides extensive analgesia on one side of the body by injecting local anesthetic into the space between the erector spinae muscle and the transverse process, allowing the anesthetic to spread into the paravertebral area through the gaps between adjacent vertebrae and block both dorsal and ventral rami of the spinal nerves.^42^^,^^43^ In contrast, the TAP block involves local anesthetic injection between the internal oblique and transversus abdominis muscles, targeting the thoracolumbar nerves and primarily managing somatic pain.^44^ The ESP block also offers additional advantages, as it is a straightforward, safe, and reliable alternative for pain relief. This ultrasound-guided technique targets the easily visualized transverse process, with the injection site located in a musculofascial plane away from the neuroaxis, pleura, and major blood vessels.^45^

Our research had several significant advantages. To begin with, we implemented an extensive search strategy that covered multiple databases. Additionally, we restricted our analysis exclusively to RCTs to ensure the credibility of our results. It is important to emphasize that our study complied with the PRISMA guidelines, demonstrating its methodological soundness. Moreover, since no prior meta-analysis has been performed on this specific topic, our study's importance is further enhanced.

Despite the valuable insights gained from our meta-analysis, it is important to recognize certain limitations in our study. Firstly, the number of articles meeting our inclusion criteria was limited, and the follow-up periods in the included studies were relatively short. Additionally, some trials did not implement blinding techniques, which introduces a potential source of bias. The scarcity of studies also prevented us from conducting subgroup analyses. We utilized TAPB as the control group and did not assess the analgesic efficacy of ESPB in comparison to other regional anesthesia techniques, highlighting a possible direction for future research and investigation in this area. The small sample sizes across the included trials may diminish statistical power and restrict the generalizability of our findings. Moreover, the observed heterogeneity in analgesic duration may affect the robustness of our results. This heterogeneity may stem from several factors, including variations in local anesthetic agents, volumes, and dosages, as well as the inclusion or exclusion of adjuvants for both blocks. Differences in spinal anesthesia protocols, with some studies using hyperbaric bupivacaine alone and others combining it with fentanyl, further contribute to this variability. Lastly, the use of different types of analgesics postoperatively across studies adds another layer of complexity.

To support our conclusions and gather additional data, it is essential to carry out more high-quality prospective randomized controlled trials. Further studies should be conducted to compare the pain-relieving effects of ESPB against TAPB and other regional anesthesia techniques in patients undergoing cesarean sections. Additionally, more research is necessary to investigate the long-term impact of ESPB on pain management and postoperative recovery related to this procedure. The effects of ESPB on pain relief for other types of transabdominal surgeries also require further examination. It is recommended that additional research should be conducted across various cultures with different socio-economic backgrounds. Furthermore, future studies should aim to thoroughly understand the mechanism behind the visceral pain relief provided by ESPB. Future research should include a cost-effectiveness analysis comparing ESPB and TAPB to determine the economic implications of these analgesic techniques.

## Conclusions

The ultrasound-guided ESP block is more effective in managing pain, reducing opioid use, providing longer-lasting pain relief, and increasing patient satisfaction following cesarean sections when compared to the TAP block group. Nonetheless, additional studies are required to verify these findings.

## Funding source

This research did not receive any specific grant from funding agencies in the public, commercial, or not-for-profit sectors.

## Conflicts of interest

The authors declare no conflicts of interest.

## References

[1] Betran A.P., Ye J., Moller A-B, Souza J.P., Zhang J. (2021). Trends and projections of caesarean section rates: global and regional estimates. BMJ Glob Health.

[2] Borges N.C., Pereira L.V., Moura L.A., Silva T.C., Pedroso CF. (2016). Predictors for Moderate to Severe Acute Postoperative Pain after Cesarean Section. Pain Res Manag.

[3] Sun K.W., Pan PH. (2019). Persistent pain after cesarean delivery. Int J Obstet Anesth.

[4] Zandomenico J.G., Perito G.Z., Machado J.A., Silva HCG. (2022). Postoperative pain management after cesarean delivery: cross-sectional study. Braz J Anesthesiol.

[5] Komatsu R., Ando K., Flood PD. (2020). Factors associated with persistent pain after childbirth: a narrative review. Br J Anaesth.

[6] Shen D., Hasegawa-Moriyama M., Ishida K., Fuseya S., Tanaka S., Kawamata M. (2020). Acute postoperative pain is correlated with the early onset of postpartum depression after cesarean section: a retrospective cohort study. J Anesth.

[7] Chang C-Y, Tu Y-K, Kao M-C (2023). Effects of opioids administered via intravenous or epidural patient-controlled analgesia after caesarean section: a network meta-analysis of randomised controlled trials. eClinicalMedicine.

[8] Holland E., Sudhof L.S., Zera C. (2020). Optimal pain management for cesarean delivery. Int Anesthesiol Clin.

[9] Aly M., Ibrahim A., Farrag W., Abdelsalam K., Mohamed H., Tawfik A. (2018). Pruritus after intrathecal morphine for cesarean delivery: incidence, severity and its relation to serum serotonin level. Int J Obstet Anesth.

[10] Zanolli N.C., Fuller M.E., Krishnamoorthy V., Ohnuma T., Raghunathan K., Habib AS. (2023). Opioid-Sparing Multimodal Analgesia Use After Cesarean Delivery Under General Anesthesia: A Retrospective Cohort Study in 729 US Hospitals. Anesth Analg.

[11] Ryu C., Choi G.J., Jung Y.H., Baek C.W., Cho C.K., Kang H. (2022). Postoperative Analgesic Effectiveness of Peripheral Nerve Blocks in Cesarean Delivery: A Systematic Review and Network Meta-Analysis. J Pers Med.

[12] Mallan D., Sharan S., Saxena S., Singh TK. (2019). Anesthetic techniques: focus on transversus abdominis plane (TAP) blocks. Local Reg Anesth.

[13] Zhao X., Tong Y., Ren H. (2014). Transversus abdominis plane block for postoperative analgesia after laparoscopic surgery: a systematic review and meta-analysis. Int J Clin Exp Med.

[14] Habib A.S., Nedeljkovic S.S., Horn J-L (2021). Randomized trial of transversus abdominis plane block with liposomal bupivacaine after cesarean delivery with or without intrathecal morphine. J Clin Anesth.

[15] Kintu A., Abdulla S., Lubikire A. (2019). Postoperative pain after cesarean section: assessment and management in a tertiary hospital in a low-income country. BMC Health Serv Res.

[16] Altıparmak B., Korkmaz Toker M., Uysal A.I., Kuşçu Y., Gümüş Demirbilek S. (2019). Ultrasound-guided erector spinae plane block versus oblique subcostal transversus abdominis plane block for postoperative analgesia of adult patients undergoing laparoscopic cholecystectomy: Randomized, controlled trial. J Clin Anesth.

[17] Jain K., Jaiswal V., Puri A. (2018). Erector spinae plane block: Relatively new block on horizon with a wide spectrum of application – A case series. Indian J Anaesth.

[18] Forero M., Adhikary S.D., Lopez H., Tsui C., Chin KJ. (2016). The Erector Spinae Plane Block: A Novel Analgesic Technique in Thoracic Neuropathic Pain. Reg Anesth Pain Med.

[19] Qi-Hong S., Xu-Yan Z., Xu S., Yan-Jun C., Ke L., Rong W. (2021). Comparison of Ultrasound-Guided Erector Spinae Plane Block and Oblique Subcostal Transverse Abdominis Plane Block for Postoperative Analgesia in Elderly Patients After Laparoscopic Colorectal Surgery: A Prospective Randomized Study. Pain Ther.

[20] Silverman M., Zwolinski N., Wang E. (2023). Regional Analgesia for Cesarean Delivery: A Narrative Review Toward Enhancing Outcomes in Parturients. J Pain Res.

[21] Aygun H., Ozturk N.K., Ugur M. (2022). Evaluation of ultrasound-guided bilateral low thoracic erector spinae plane block for postoperative analgesia in cesarean delivery patients: a prospective, randomized, controlled clinical trial. Braz J Anesthesiol Engl Ed.

[22] Malawat A., Verma K., Jethava D., Jethava DD. (2020). Erector spinae plane block and transversus abdominis plane block for postoperative analgesia in cesarean section: A prospective randomized comparative study. J Anaesthesiol Clin Pharmacol.

[23] Boules M.L., Goda A.S., Abdelhady M.A., Abu El-Nour Abd El-Azeem S.A., Hamed M.A. (2020). Comparison of Analgesic Effect Between Erector Spinae Plane Block and Transversus Abdominis Plane Block After Elective Cesarean Section: A Prospective Randomized Single-Blind Controlled Study. J Pain Res.

[24] Eksteen A., Wagner J., Kleyenstuber T., Kamerman P. (2024). Comparison of erector spinae plane and transversus abdominis plane blocks for postoperative analgesia after caesarean delivery under spinal anaesthesia: a randomised controlled trial. Int J Obstet Anesth.

[25] Reddy N.V.K., Prabhu M., Kanakalakshmi S.T., Muhamed S. (2023). Randomized comparison between transversus abdominis and erector spinae blocks in cesarean section. Colomb J Anesthesiol.

[26] Moher D., Liberati A., Tetzlaff J., Altman DG. (2009). PRISMA Group Preferred reporting items for systematic reviews and meta-analyses: the PRISMA statement. PLoS Med.

[27] Wan X., Wang W., Liu J., Tong T. (2014). Estimating the sample mean and standard deviation from the sample size, median, range and/or interquartile range. BMC Med Res Methodol.

[28] Higgins J.P.T., Altman D.G., Gøtzsche P.C. (2011). The Cochrane Collaboration's tool for assessing risk of bias in randomised trials. BMJ.

[29] Atkins D., Eccles M., Flottorp S. (2004). Systems for grading the quality of evidence and the strength of recommendations I: critical appraisal of existing approaches The GRADE Working Group. BMC Health Serv Res.

[30] Wasserstein R.L., Lazar NA. (2016). The ASA Statement on p-Values: Context, Process, and Purpose. Am Stat.

[31] Julian T Higgins AP, Thompson SG, Deeks JJ. Measuring inconsistency in meta-analyses Rapid responses Topic collections. 2003;327:557-60. 10.1136/bmj.327.7414.557 PMC192859 12958120

[32] Egger M., Smith GD., Schneider M., Minder C. (2015). Bias in meta­analysis detected by a simple, graphical test. BMJ.

[33] Terrin N., Schmid C.H., Lau J., Olkin I. (2003). Adjusting for publication bias in the presence of heterogeneity. Stat Med Stat Med.

[34] Balata A.A.A., El-Dorgham L.T., Ismai H.A.M., El-Dorgham L.T. (2023). Analgesic Efficacy of Ultrasound-Guided Erector Spinae Plane Block versus Transversus Abdominis Plane Block for Post-cesarean Delivery Pain under Spinal Anesthesia. Egypt J Hosp Med.

[35] Elshafay A., Hegazy E.M., Ashour F.H. (2022). Abd-Almoniem MF. Analgesic Effect of Erector Spinae Plane Block versus Transversus Abdominis Plane Block After Elective Cesarean Section. Al-Azhar Univ J Med Virus Res Stud.

[36] Yilmaz M.A., Erol MK. (2023). Comparison of The Effects of Erector Spina Plane Block and Transversus Abdominis Plane Block Methods on Postoperative Analgesia in Elective Caesarian Section with Ultrasonography Account. Int J Curr Med Biol Sci.

[37] VR Junior I do, Carvalho V.H., Brito LGO. (2022). Erector spinae plane block for analgesia after cesarean delivery: a systematic review with meta-analysis. Braz J Anesthesiol.

[38] Hamed M.A., Yassin H.M., Botros J.M., Abdelhady MA. (2020). Analgesic Efficacy of Erector Spinae Plane Block Compared with Intrathecal Morphine After Elective Cesarean Section: A Prospective Randomized Controlled Study. J Pain Res.

[39] Zanfini B.A., Di Muro M., Biancone M. (2023). Ultrasound-Guided Bilateral Erector Spinae Plane Block vs. Ultrasound-Guided Bilateral Posterior Quadratus Lumborum Block for Postoperative Analgesia after Caesarean Section: An Observational Closed Mixed Cohort Study. J Clin Med.

[40] Bakshi A., Srivastawa S., Jadon A., Mohsin K., Sinha N., Chakraborty S. (2022). Comparison of the analgesic efficacy of ultrasound-guided transmuscular quadratus lumborum block versus thoracic erector spinae block for postoperative analgesia in caesarean section parturients under spinal anaesthesia ‒ A randomised study. Indian J Anaesth.

[41] Joshi R., Jeevan R., Amutha S.V., Ramakrishnan L., Natarajan NR. (2024). Comparison of ultrasound-guided erector spinae plane block versus transmuscular quadratus lumborum block for postoperative analgesia after caesarean delivery: A prospective randomized non-inferiority clinical trial. J Anaesthesiol Clin Pharmacol.

[42] Saadawi M., Layera S., Aliste J., Bravo D., Leurcharusmee P., Tran DQ. (2021). Erector spinae plane block: A narrative review with systematic analysis of the evidence pertaining to clinical indications and alternative truncal blocks. J Clin Anesth.

[43] Ueshima H., Otake H. (2017). Similarities Between the Retrolaminar and Erector Spinae Plane Blocks. Reg Anesth Pain Med.

[44] Tsai H-C, Yoshida T., Chuang T-Y (2017). Transversus Abdominis Plane Block: An Updated Review of Anatomy and Techniques. BioMed Res Int.

[45] Kot P., Rodriguez P., Granell M. (2019). The erector spinae plane block: a narrative review. Korean J Anesthesiol.

